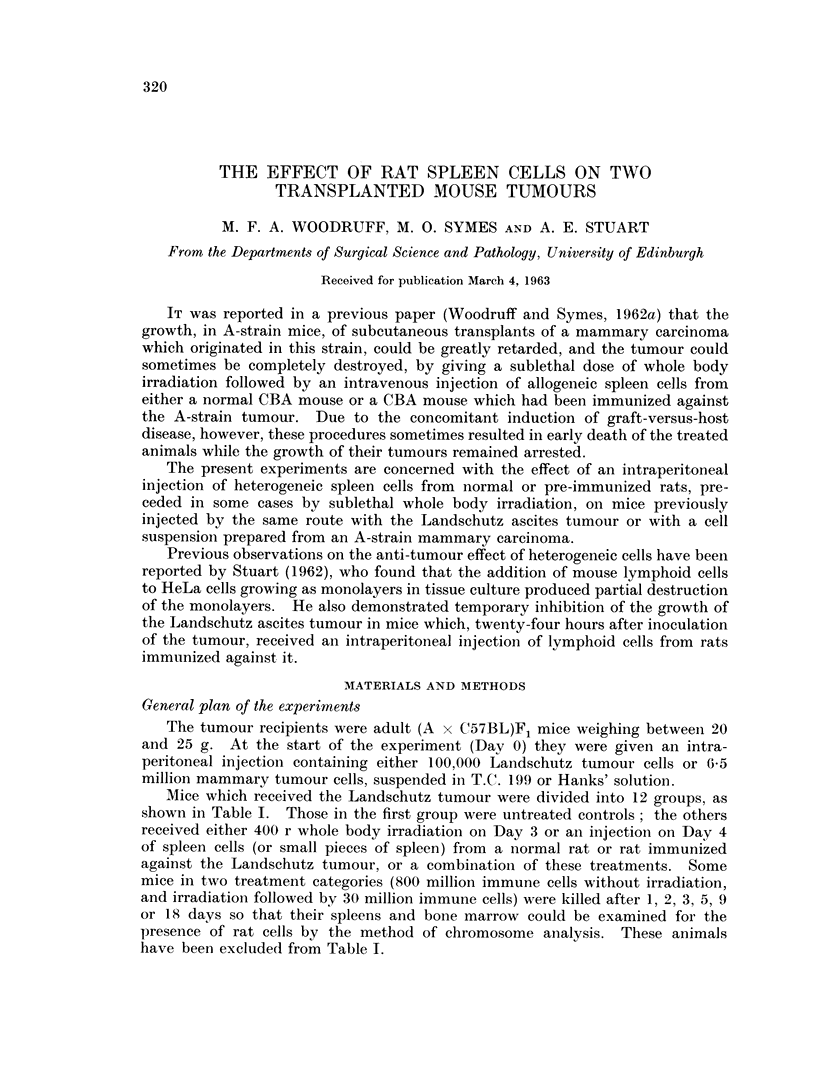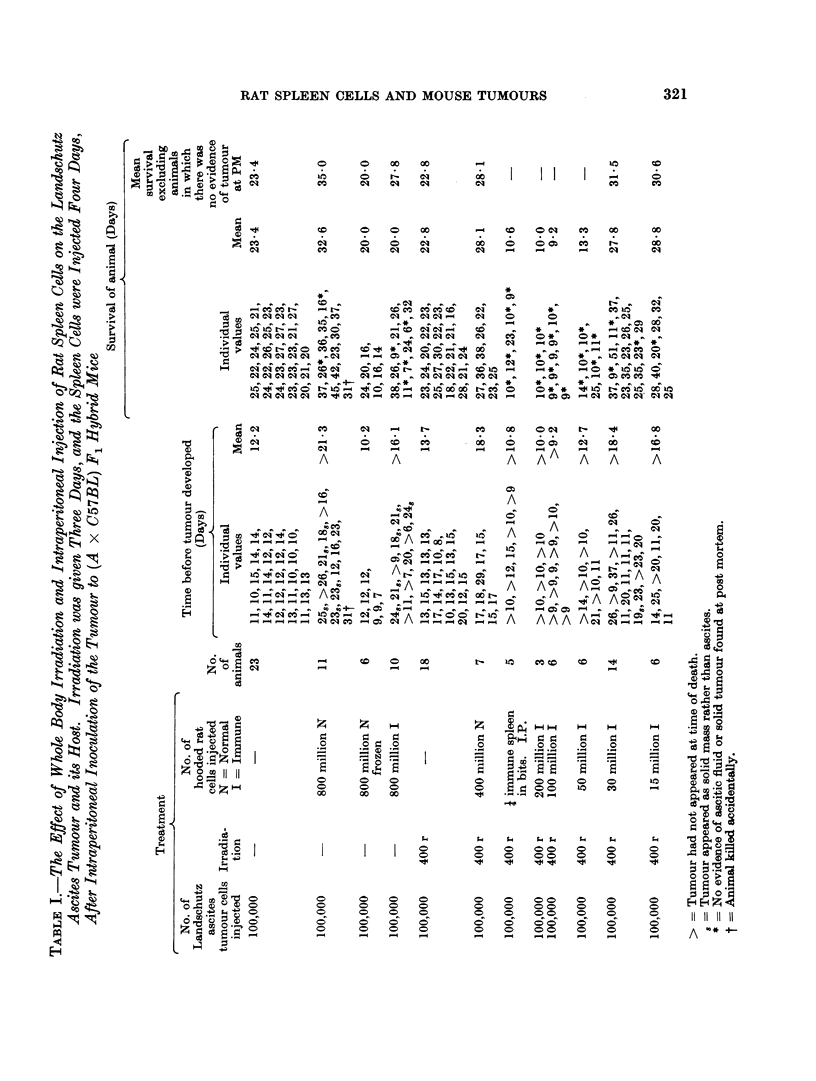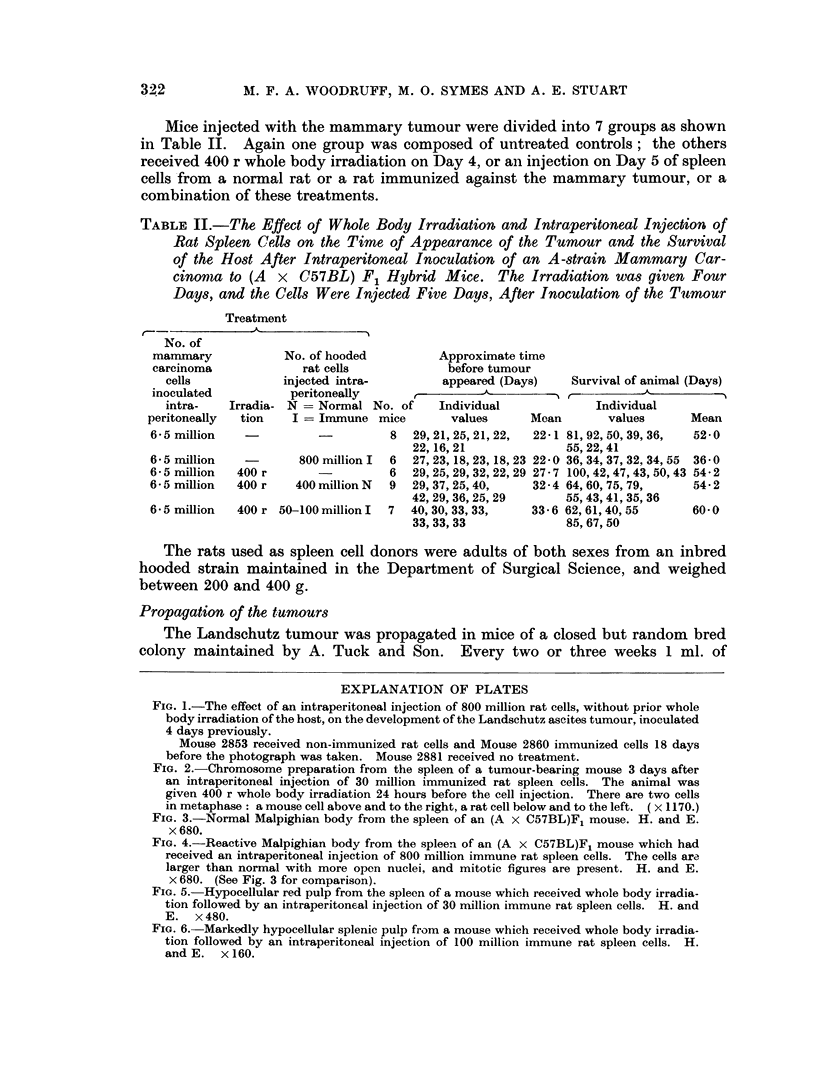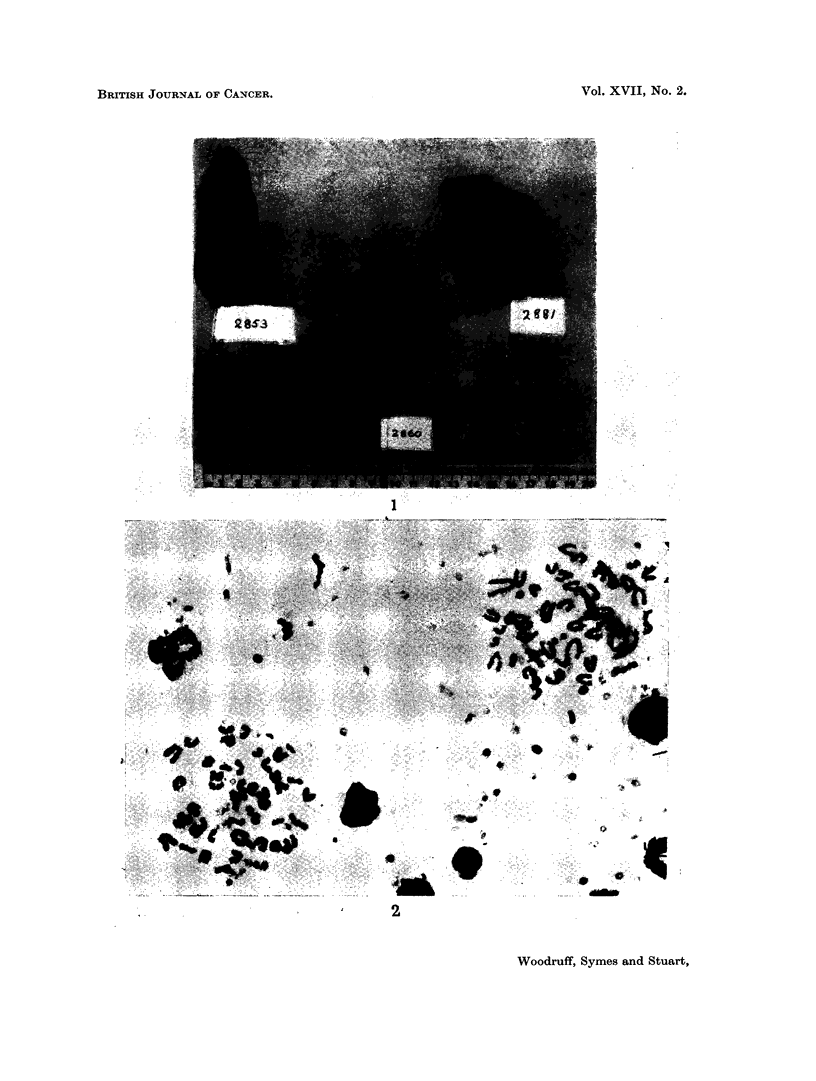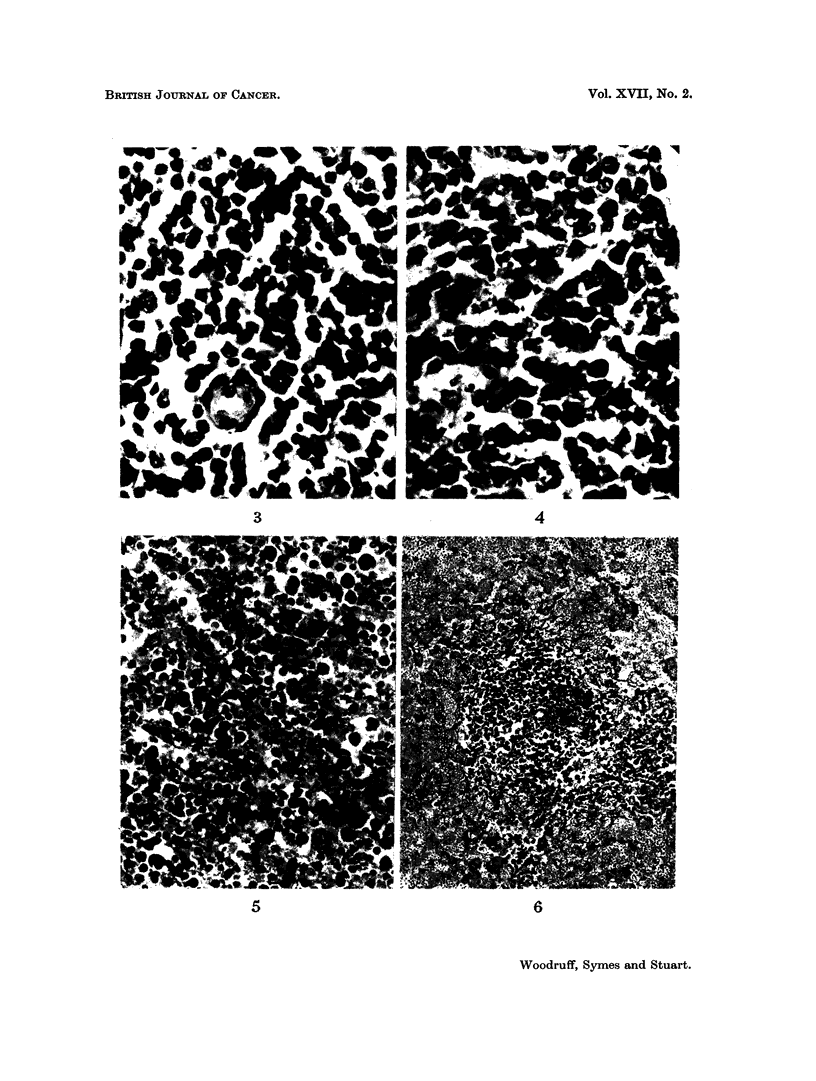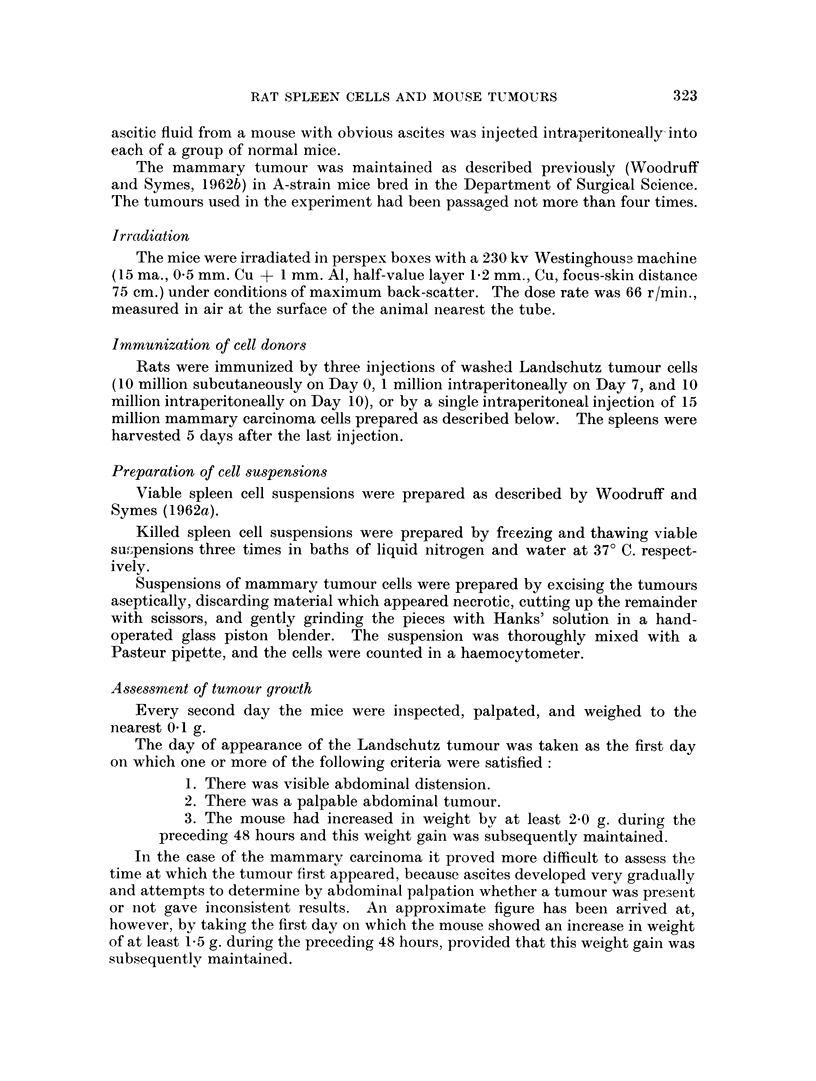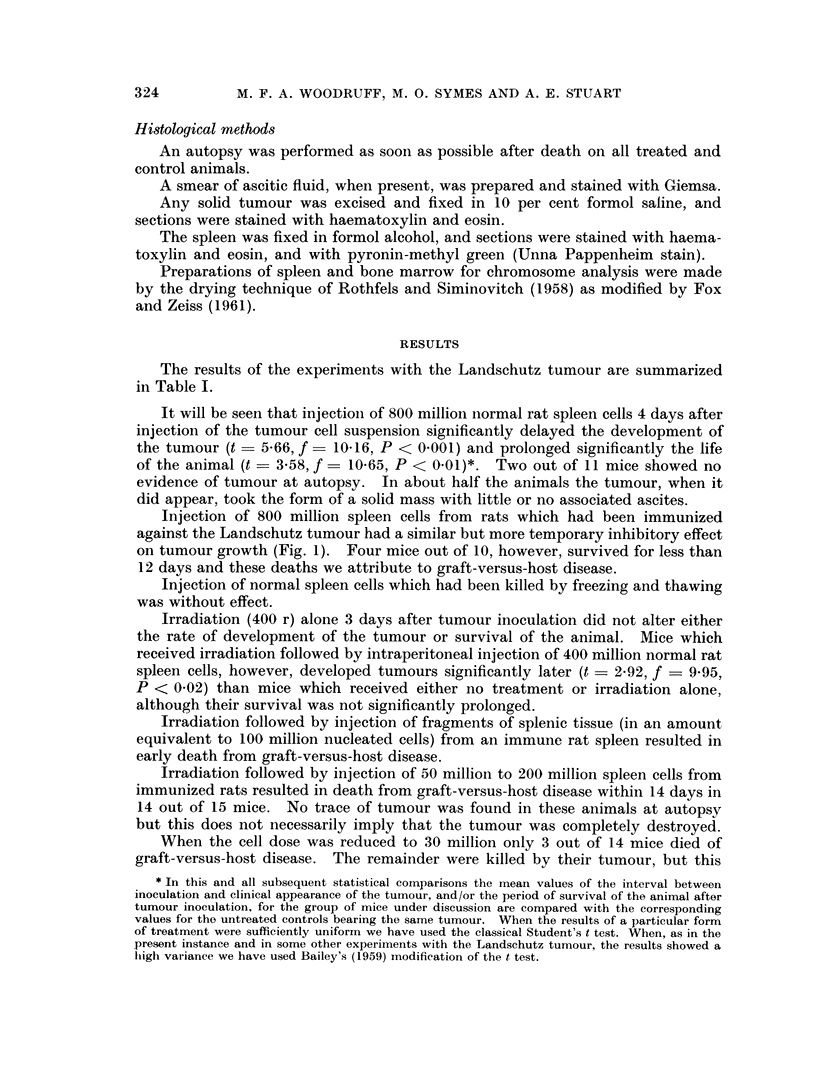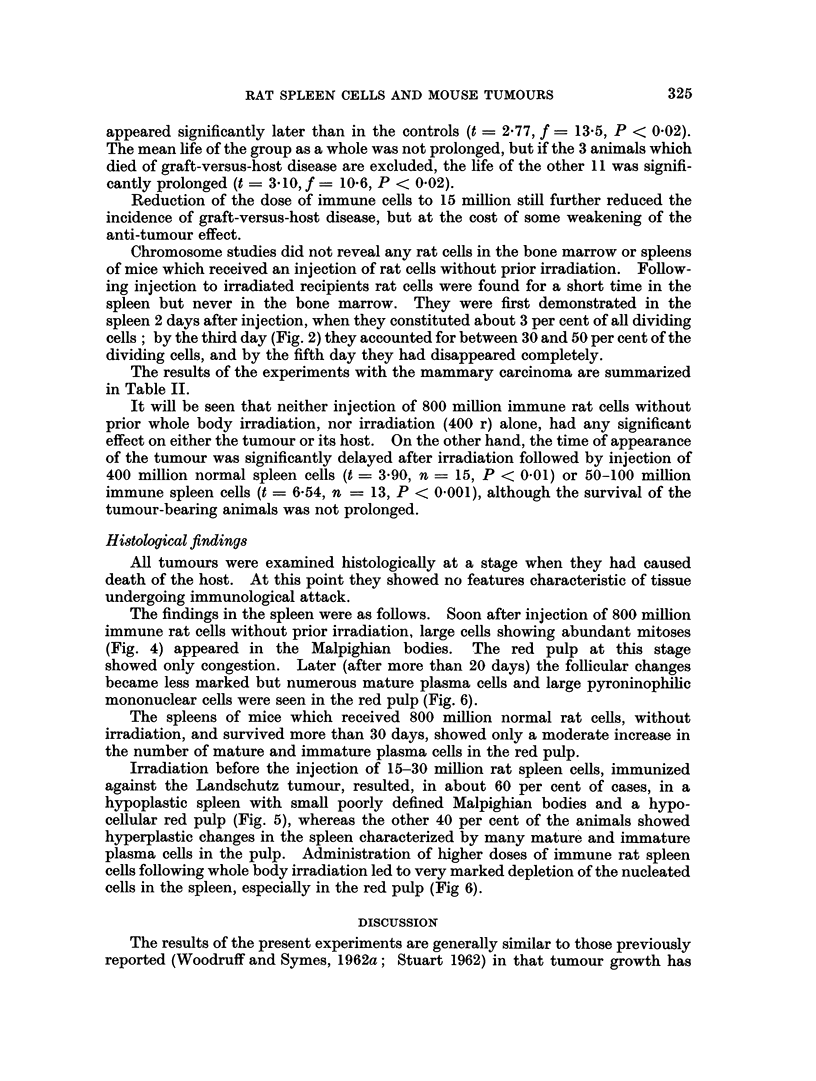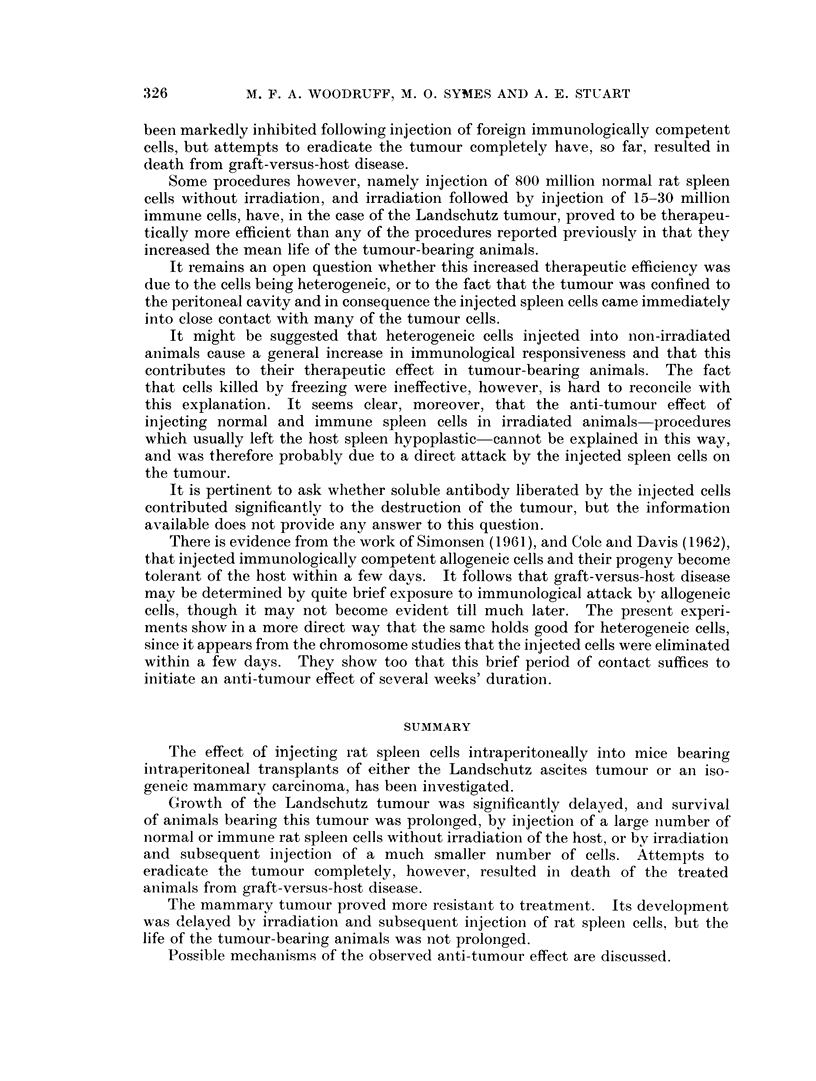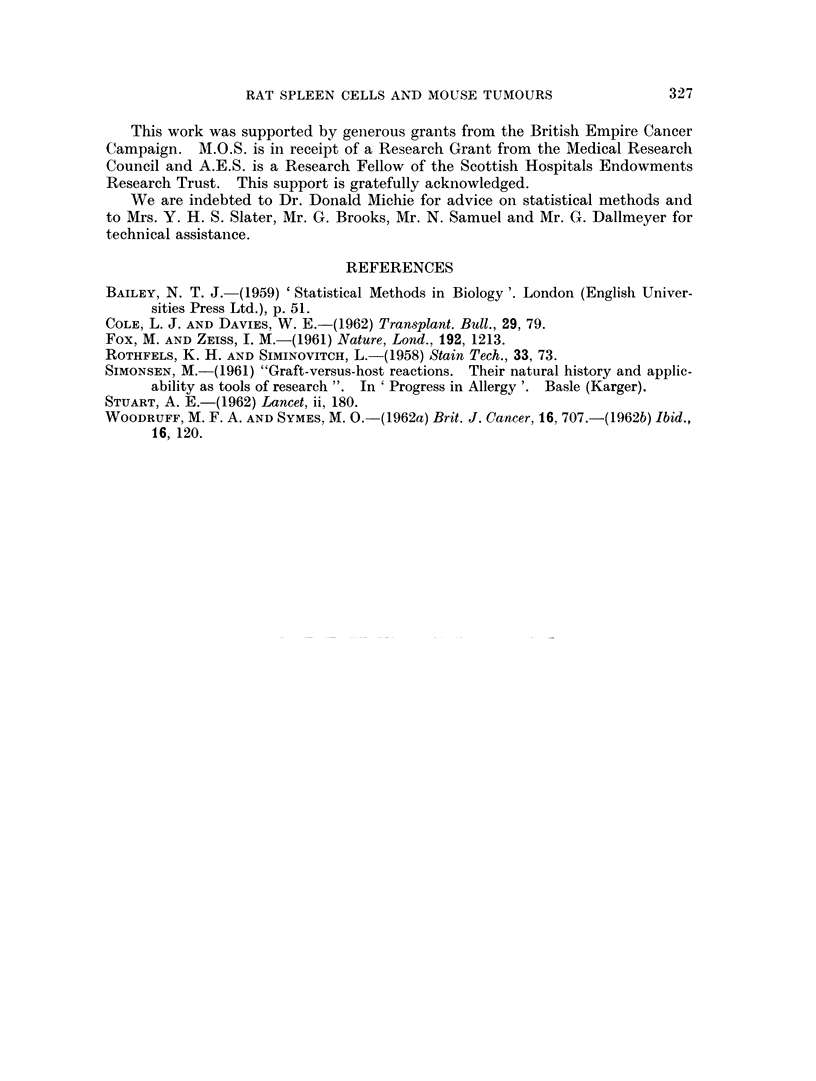# The Effect of Rat Spleen Cells on Two Transplanted Mouse Tumours

**DOI:** 10.1038/bjc.1963.46

**Published:** 1963-06

**Authors:** M. F. A. Woodruff, M. O. Symes, A. E. Stuart

## Abstract

**Images:**


### THE EFFECT OF RAT SPLEEN CELLS ON TWO TRANSPLANTED MOUSE TUMOURS

M. F. A. WOODRUFF, M. O. SYMES AND A. E. STUART

*From the Departments of Surgical Science and Pathology, University of Edinburgh*

Received for publication March 4, 1963

It was reported in a previous paper (Woodruff and Symes, 1962*a*) that the growth, in A-strain mice, of subcutaneous transplants of a mammary carcinoma which originated in this strain, could be greatly retarded, and the tumour could sometimes be completely destroyed, by giving a sublethal dose of whole body irradiation followed by an intravenous injection of allogeneic spleen cells from either a normal CBA mouse or a CBA mouse which had been immunized against the A-strain tumour. Due to the concomitant induction of graft-versus-host disease, however, these procedures sometimes resulted in early death of the treated animals while the growth of their tumours remained arrested.

The present experiments are concerned with the effect of an intraperitoneal injection of heterogeneic spleen cells from normal or pre-immunized rats, preceded in some cases by sublethal whole body irradiation, on mice previously injected by the same route with the Landschutz ascites tumour or with a cell suspension prepared from an A-strain mammary carcinoma.

Previous observations on the anti-tumour effect of heterogeneic cells have been reported by Stuart (1962), who found that the addition of mouse lymphoid cells to HeLa cells growing as monolayers in tissue culture produced partial destruction of the monolayers. He also demonstrated temporary inhibition of the growth of the Landschutz ascites tumour in mice which, twenty-four hours after inoculation of the tumour, received an intraperitoneal injection of lymphoid cells from rats immunized against it.

### MATERIALS AND METHODS

### *General plan of the experiments*

The tumour recipients were adult (A × C57BL)$F_1$ mice weighing between 20 and 25 g. At the start of the experiment (Day 0) they were given an intraperitoneal injection containing either 100,000 Landschutz tumour cells or 6·5 million mammary tumour cells, suspended in T.C. 199 or Hanks' solution.

Mice which received the Landschutz tumour were divided into 12 groups, as shown in Table I. Those in the first group were untreated controls ; the others received either 400 r whole body irradiation on Day 3 or an injection on Day 4 of spleen cells (or small pieces of spleen) from a normal rat or rat immunized against the Landschutz tumour, or a combination of these treatments. Some mice in two treatment categories (800 million immune cells without irradiation, and irradiation followed by 30 million immune cells) were killed after 1, 2, 3, 5, 9 or 18 days so that their spleens and bone marrow could be examined for the presence of rat cells by the method of chromosome analysis. These animals have been excluded from Table I.

Table I.—*The Effect of Whole Body Irradiation and Intraperitoneal Injection of Rat Spleen Cells on the Landschutz Ascites Tumour and its Host. Irradiation was given Three Days, and the Spleen Cells were Injected Four Days, After Intraperitoneal Inoculation of the Tumour to (A × C57BL) $F_1$ Hybrid Mice*

| Treatment | | | | Time before tumour developed (Days) | | Survival of animal (Days) | | |
|---|---|---|---|---|---|---|---|---|
| No. of Landschutz ascites tumour cells injected | Irradiation | No. of hooded rat cells injected N = Normal I = Immune | No. of animals | Individual values | Mean | Individual values | Mean | Mean survival excluding animals in which there was no evidence of tumour at PM |
| 100,000 | — | — | 23 | 11, 10, 15, 14, 14, 14, 11, 14, 12, 12, 12, 12, 12, 12, 14, 13, 11, 10, 10, 10, 11, 13, 13 | 12·2 | 25, 22, 24, 25, 21, 24, 22, 26, 25, 23, 24, 23, 27, 27, 23, 23, 23, 23, 21, 27, 20, 21, 20 | 23·4 | 23·4 |
| 100,000 | — | 800 million N | 11 | $25_s$, >26, $21_s$, $18_s$, >16, $23_s$, $23_s$, 12, 16, 23, 31† | >21·3 | 37, 26*, 36, 35, 16*, 45, 42, 23, 30, 37, 31† | 32·6 | 35·0 |
| 100,000 | — | 800 million N frozen | 6 | 12, 12, 12, 9, 9, 7 | 10·2 | 24, 20, 16, 10, 16, 14 | 20·0 | 20·0 |
| 100,000 | — | 800 million I | 10 | $24_s$, $21_s$, >9, $18_s$, $21_s$, >11, >7, 20, >6, $24_s$ | >16·1 | 38, 26, 9*, 21, 26, 11*, 7*, 24, 6*, 32 | 20·0 | 27·8 |
| 100,000 | 400 r | — | 18 | 13, 15, 13, 13, 13, 17, 14, 17, 10, 8, 10, 13, 15, 13, 15, 20, 12, 15 | 13·7 | 23, 24, 20, 22, 23, 25, 27, 30, 22, 23, 18, 22, 21, 21, 16, 28, 21, 24 | 22·8 | 22·8 |
| 100,000 | 400 r | 400 million N | 7 | 17, 18, 29, 17, 15, 15, 17 | 18·3 | 27, 36, 38, 26, 22, 23, 25 | 28·1 | 28·1 |
| 100,000 | 400 r | ¼ immune spleen in bits. I.P. | 5 | >10, >12, 15, >10, >9 | >10·8 | 10*, 12*, 23, 10*, 9* | 10·6 | — |
| 100,000 | 400 r | 200 million I | 3 | >10, >10, >10 | >10·0 | 10*, 10*, 10* | 10·0 | — |
| 100,000 | 400 r | 100 million I | 6 | >9, >9, 9, >9, >10, >9 | >9·2 | 9*, 9*, 9, 9*, 10*, 9* | 9·2 | — |
| 100,000 | 400 r | 50 million I | 6 | >14, >10, >10, 21, >10, 11 | >12·7 | 14*, 10*, 10*, 25, 10*, 11* | 13·3 | — |
| 100,000 | 400 r | 30 million I | 14 | 26, >9, 37, >11, 26, 11, 20, 11, 11, 11, $19_s$, 23, >23, 20 | >18·4 | 37, 9*, 51, 11*, 37, 23, 35, 23, 26, 25, 25, 35, 23*, 29 | 27·8 | 31·5 |
| 100,000 | 400 r | 15 million I | 6 | 14, 25, >20, 11, 20, 11 | >16·8 | 28, 40, 20*, 28, 32, 25 | 28·8 | 30·6 |

> = Tumour had not appeared at time of death.

$_s$ = Tumour appeared as solid mass rather than ascites.

* = No evidence of ascitic fluid or solid tumour found at post mortem.

† = Animal killed accidentally.

Mice injected with the mammary tumour were divided into 7 groups as shown in Table II. Again one group was composed of untreated controls; the others received 400 r whole body irradiation on Day 4, or an injection on Day 5 of spleen cells from a normal rat or a rat immunized against the mammary tumour, or a combination of these treatments.

TABLE II.—*The Effect of Whole Body Irradiation and Intraperitoneal Injection of Rat Spleen Cells on the Time of Appearance of the Tumour and the Survival of the Host After Intraperitoneal Inoculation of an A-strain Mammary Carcinoma to (A × C57BL) $F_1$ Hybrid Mice. The Irradiation was given Four Days, and the Cells Were Injected Five Days, After Inoculation of the Tumour*

| Treatment | | | | Approximate time before tumour appeared (Days) | | Survival of animal (Days) | |
|---|---|---|---|---|---|---|---|
| No. of mammary carcinoma cells inoculated intra-peritoneally | Irradiation | No. of hooded rat cells injected intra-peritoneally N = Normal I = Immune | No. of mice | Individual values | Mean | Individual values | Mean |
| 6·5 million | — | — | 8 | 29, 21, 25, 21, 22, 22, 16, 21 | 22·1 | 81, 92, 50, 39, 36, 55, 22, 41 | 52·0 |
| 6·5 million | — | 800 million I | 6 | 27, 23, 18, 23, 18, 23 | 22·0 | 36, 34, 37, 32, 34, 55 | 36·0 |
| 6·5 million | 400 r | — | 6 | 29, 25, 29, 32, 22, 29 | 27·7 | 100, 42, 47, 43, 50, 43 | 54·2 |
| 6·5 million | 400 r | 400 million N | 9 | 29, 37, 25, 40, 42, 29, 36, 25, 29 | 32·4 | 64, 60, 75, 79, 55, 43, 41, 35, 36 | 54·2 |
| 6·5 million | 400 r | 50–100 million I | 7 | 40, 30, 33, 33, 33, 33, 33 | 33·6 | 62, 61, 40, 55 85, 67, 50 | 60·0 |

The rats used as spleen cell donors were adults of both sexes from an inbred hooded strain maintained in the Department of Surgical Science, and weighed between 200 and 400 g.

*Propagation of the tumours*

The Landschutz tumour was propagated in mice of a closed but random bred colony maintained by A. Tuck and Son. Every two or three weeks 1 ml. of

EXPLANATION OF PLATES

FIG. 1.—The effect of an intraperitoneal injection of 800 million rat cells, without prior whole body irradiation of the host, on the development of the Landschutz ascites tumour, inoculated 4 days previously.

Mouse 2853 received non-immunized rat cells and Mouse 2860 immunized cells 18 days before the photograph was taken. Mouse 2881 received no treatment.

FIG. 2.—Chromosome preparation from the spleen of a tumour-bearing mouse 3 days after an intraperitoneal injection of 30 million immunized rat spleen cells. The animal was given 400 r whole body irradiation 24 hours before the cell injection. There are two cells in metaphase: a mouse cell above and to the right, a rat cell below and to the left. (×1170.)

FIG. 3.—Normal Malpighian body from the spleen of an (A × C57BL)$F_1$ mouse. H. and E. ×680.

FIG. 4.—Reactive Malpighian body from the spleen of an (A × C57BL)$F_1$ mouse which had received an intraperitoneal injection of 800 million immune rat spleen cells. The cells are larger than normal with more open nuclei, and mitotic figures are present. H. and E. ×680. (See Fig. 3 for comparison).

FIG. 5.—Hypocellular red pulp from the spleen of a mouse which received whole body irradiation followed by an intraperitoneal injection of 30 million immune rat spleen cells. H. and E. ×480.

FIG. 6.—Markedly hypocellular splenic pulp from a mouse which received whole body irradiation followed by an intraperitoneal injection of 100 million immune rat spleen cells. H. and E. ×160.

1

2

Woodruff, Symes and Stuart,

3

4

5

6

ascitic fluid from a mouse with obvious ascites was injected intraperitoneally into each of a group of normal mice.

The mammary tumour was maintained as described previously (Woodruff and Symes, 1962*b*) in A-strain mice bred in the Department of Surgical Science. The tumours used in the experiment had been passaged not more than four times.

*Irradiation*

The mice were irradiated in perspex boxes with a 230 kv Westinghouse machine (15 ma., 0·5 mm. Cu + 1 mm. Al, half-value layer 1·2 mm., Cu, focus-skin distance 75 cm.) under conditions of maximum back-scatter. The dose rate was 66 r/min., measured in air at the surface of the animal nearest the tube.

*Immunization of cell donors*

Rats were immunized by three injections of washed Landschutz tumour cells (10 million subcutaneously on Day 0, 1 million intraperitoneally on Day 7, and 10 million intraperitoneally on Day 10), or by a single intraperitoneal injection of 15 million mammary carcinoma cells prepared as described below. The spleens were harvested 5 days after the last injection.

*Preparation of cell suspensions*

Viable spleen cell suspensions were prepared as described by Woodruff and Symes (1962*a*).

Killed spleen cell suspensions were prepared by freezing and thawing viable suspensions three times in baths of liquid nitrogen and water at 37° C. respectively.

Suspensions of mammary tumour cells were prepared by excising the tumours aseptically, discarding material which appeared necrotic, cutting up the remainder with scissors, and gently grinding the pieces with Hanks' solution in a hand-operated glass piston blender. The suspension was thoroughly mixed with a Pasteur pipette, and the cells were counted in a haemocytometer.

*Assessment of tumour growth*

Every second day the mice were inspected, palpated, and weighed to the nearest 0·1 g.

The day of appearance of the Landschutz tumour was taken as the first day on which one or more of the following criteria were satisfied :

1. There was visible abdominal distension.
2. There was a palpable abdominal tumour.
3. The mouse had increased in weight by at least 2·0 g. during the preceding 48 hours and this weight gain was subsequently maintained.

In the case of the mammary carcinoma it proved more difficult to assess the time at which the tumour first appeared, because ascites developed very gradually and attempts to determine by abdominal palpation whether a tumour was present or not gave inconsistent results. An approximate figure has been arrived at, however, by taking the first day on which the mouse showed an increase in weight of at least 1·5 g. during the preceding 48 hours, provided that this weight gain was subsequently maintained.

*Histological methods*

An autopsy was performed as soon as possible after death on all treated and control animals.

A smear of ascitic fluid, when present, was prepared and stained with Giemsa.

Any solid tumour was excised and fixed in 10 per cent formol saline, and sections were stained with haematoxylin and eosin.

The spleen was fixed in formol alcohol, and sections were stained with haematoxylin and eosin, and with pyronin-methyl green (Unna Pappenheim stain).

Preparations of spleen and bone marrow for chromosome analysis were made by the drying technique of Rothfels and Siminovitch (1958) as modified by Fox and Zeiss (1961).

### RESULTS

The results of the experiments with the Landschutz tumour are summarized in Table I.

It will be seen that injection of 800 million normal rat spleen cells 4 days after injection of the tumour cell suspension significantly delayed the development of the tumour ($t = 5{\cdot}66$, $f = 10{\cdot}16$, $P < 0{\cdot}001$) and prolonged significantly the life of the animal ($t = 3{\cdot}58$, $f = 10{\cdot}65$, $P < 0{\cdot}01$)*. Two out of 11 mice showed no evidence of tumour at autopsy. In about half the animals the tumour, when it did appear, took the form of a solid mass with little or no associated ascites.

Injection of 800 million spleen cells from rats which had been immunized against the Landschutz tumour had a similar but more temporary inhibitory effect on tumour growth (Fig. 1). Four mice out of 10, however, survived for less than 12 days and these deaths we attribute to graft-versus-host disease.

Injection of normal spleen cells which had been killed by freezing and thawing was without effect.

Irradiation (400 r) alone 3 days after tumour inoculation did not alter either the rate of development of the tumour or survival of the animal. Mice which received irradiation followed by intraperitoneal injection of 400 million normal rat spleen cells, however, developed tumours significantly later ($t = 2{\cdot}92$, $f = 9{\cdot}95$, $P < 0{\cdot}02$) than mice which received either no treatment or irradiation alone, although their survival was not significantly prolonged.

Irradiation followed by injection of fragments of splenic tissue (in an amount equivalent to 100 million nucleated cells) from an immune rat spleen resulted in early death from graft-versus-host disease.

Irradiation followed by injection of 50 million to 200 million spleen cells from immunized rats resulted in death from graft-versus-host disease within 14 days in 14 out of 15 mice. No trace of tumour was found in these animals at autopsy but this does not necessarily imply that the tumour was completely destroyed.

When the cell dose was reduced to 30 million only 3 out of 14 mice died of graft-versus-host disease. The remainder were killed by their tumour, but this

* In this and all subsequent statistical comparisons the mean values of the interval between inoculation and clinical appearance of the tumour, and/or the period of survival of the animal after tumour inoculation, for the group of mice under discussion are compared with the corresponding values for the untreated controls bearing the same tumour. When the results of a particular form of treatment were sufficiently uniform we have used the classical Student's $t$ test. When, as in the present instance and in some other experiments with the Landschutz tumour, the results showed a high variance we have used Bailey's (1959) modification of the $t$ test.

appeared significantly later than in the controls ($t = 2{\cdot}77$, $f = 13{\cdot}5$, $P < 0{\cdot}02$). The mean life of the group as a whole was not prolonged, but if the 3 animals which died of graft-versus-host disease are excluded, the life of the other 11 was significantly prolonged ($t = 3{\cdot}10$, $f = 10{\cdot}6$, $P < 0{\cdot}02$).

Reduction of the dose of immune cells to 15 million still further reduced the incidence of graft-versus-host disease, but at the cost of some weakening of the anti-tumour effect.

Chromosome studies did not reveal any rat cells in the bone marrow or spleens of mice which received an injection of rat cells without prior irradiation. Following injection to irradiated recipients rat cells were found for a short time in the spleen but never in the bone marrow. They were first demonstrated in the spleen 2 days after injection, when they constituted about 3 per cent of all dividing cells ; by the third day (Fig. 2) they accounted for between 30 and 50 per cent of the dividing cells, and by the fifth day they had disappeared completely.

The results of the experiments with the mammary carcinoma are summarized in Table II.

It will be seen that neither injection of 800 million immune rat cells without prior whole body irradiation, nor irradiation (400 r) alone, had any significant effect on either the tumour or its host. On the other hand, the time of appearance of the tumour was significantly delayed after irradiation followed by injection of 400 million normal spleen cells ($t = 3{\cdot}90$, $n = 15$, $P < 0{\cdot}01$) or 50–100 million immune spleen cells ($t = 6{\cdot}54$, $n = 13$, $P < 0{\cdot}001$), although the survival of the tumour-bearing animals was not prolonged.

*Histological findings*

All tumours were examined histologically at a stage when they had caused death of the host. At this point they showed no features characteristic of tissue undergoing immunological attack.

The findings in the spleen were as follows. Soon after injection of 800 million immune rat cells without prior irradiation, large cells showing abundant mitoses (Fig. 4) appeared in the Malpighian bodies. The red pulp at this stage showed only congestion. Later (after more than 20 days) the follicular changes became less marked but numerous mature plasma cells and large pyroninophilic mononuclear cells were seen in the red pulp (Fig. 6).

The spleens of mice which received 800 million normal rat cells, without irradiation, and survived more than 30 days, showed only a moderate increase in the number of mature and immature plasma cells in the red pulp.

Irradiation before the injection of 15–30 million rat spleen cells, immunized against the Landschutz tumour, resulted, in about 60 per cent of cases, in a hypoplastic spleen with small poorly defined Malpighian bodies and a hypocellular red pulp (Fig. 5), whereas the other 40 per cent of the animals showed hyperplastic changes in the spleen characterized by many mature and immature plasma cells in the pulp. Administration of higher doses of immune rat spleen cells following whole body irradiation led to very marked depletion of the nucleated cells in the spleen, especially in the red pulp (Fig 6).

### DISCUSSION

The results of the present experiments are generally similar to those previously reported (Woodruff and Symes, 1962*a* ; Stuart 1962) in that tumour growth has been markedly inhibited following injection of foreign immunologically competent cells, but attempts to eradicate the tumour completely have, so far, resulted in death from graft-versus-host disease.

Some procedures however, namely injection of 800 million normal rat spleen cells without irradiation, and irradiation followed by injection of 15–30 million immune cells, have, in the case of the Landschutz tumour, proved to be therapeutically more efficient than any of the procedures reported previously in that they increased the mean life of the tumour-bearing animals.

It remains an open question whether this increased therapeutic efficiency was due to the cells being heterogeneic, or to the fact that the tumour was confined to the peritoneal cavity and in consequence the injected spleen cells came immediately into close contact with many of the tumour cells.

It might be suggested that heterogeneic cells injected into non-irradiated animals cause a general increase in immunological responsiveness and that this contributes to their therapeutic effect in tumour-bearing animals. The fact that cells killed by freezing were ineffective, however, is hard to reconcile with this explanation. It seems clear, moreover, that the anti-tumour effect of injecting normal and immune spleen cells in irradiated animals—procedures which usually left the host spleen hypoplastic—cannot be explained in this way, and was therefore probably due to a direct attack by the injected spleen cells on the tumour.

It is pertinent to ask whether soluble antibody liberated by the injected cells contributed significantly to the destruction of the tumour, but the information available does not provide any answer to this question.

There is evidence from the work of Simonsen (1961), and Cole and Davis (1962), that injected immunologically competent allogeneic cells and their progeny become tolerant of the host within a few days. It follows that graft-versus-host disease may be determined by quite brief exposure to immunological attack by allogeneic cells, though it may not become evident till much later. The present experiments show in a more direct way that the same holds good for heterogeneic cells, since it appears from the chromosome studies that the injected cells were eliminated within a few days. They show too that this brief period of contact suffices to initiate an anti-tumour effect of several weeks' duration.

### SUMMARY

The effect of injecting rat spleen cells intraperitoneally into mice bearing intraperitoneal transplants of either the Landschutz ascites tumour or an isogeneic mammary carcinoma, has been investigated.

Growth of the Landschutz tumour was significantly delayed, and survival of animals bearing this tumour was prolonged, by injection of a large number of normal or immune rat spleen cells without irradiation of the host, or by irradiation and subsequent injection of a much smaller number of cells. Attempts to eradicate the tumour completely, however, resulted in death of the treated animals from graft-versus-host disease.

The mammary tumour proved more resistant to treatment. Its development was delayed by irradiation and subsequent injection of rat spleen cells, but the life of the tumour-bearing animals was not prolonged.

Possible mechanisms of the observed anti-tumour effect are discussed.

This work was supported by generous grants from the British Empire Cancer Campaign. M.O.S. is in receipt of a Research Grant from the Medical Research Council and A.E.S. is a Research Fellow of the Scottish Hospitals Endowments Research Trust. This support is gratefully acknowledged.

We are indebted to Dr. Donald Michie for advice on statistical methods and to Mrs. Y. H. S. Slater, Mr. G. Brooks, Mr. N. Samuel and Mr. G. Dallmeyer for technical assistance.

### REFERENCES

BAILEY, N. T. J.—(1959) ' Statistical Methods in Biology '. London (English Universities Press Ltd.), p. 51.

COLE, L. J. AND DAVIES, W. E.—(1962) *Transplant. Bull.*, **29**, 79.

FOX, M. AND ZEISS, I. M.—(1961) *Nature, Lond.*, **192**, 1213.

ROTHFELS, K. H. AND SIMINOVITCH, L.—(1958) *Stain Tech.*, **33**, 73.

SIMONSEN, M.—(1961) "Graft-versus-host reactions. Their natural history and applicability as tools of research ". In ' Progress in Allergy '. Basle (Karger).

STUART, A. E.—(1962) *Lancet*, ii, 180.

WOODRUFF, M. F. A. AND SYMES, M. O.—(1962*a*) *Brit. J. Cancer*, **16**, 707.—(1962*b*) *Ibid.*, **16**, 120.